# Supplementary material for: Inter- and intracellular colonization of Arabidopsis roots by endophytic actinobacteria and the impact of plant hormones on their antimicrobial activity
Source: Antonie Van Leeuwenhoek. 2018 Jan 15;111(5):679–90. doi: 10.1007/s10482-018-1014-z (PMC5913384; doi:10.1007/s10482-018-1014-z)
Supplement: Supplementary file 1 — Supplementary material 1 (PDF 796 kb) [file 10482_2018_1014_MOESM1_ESM.pdf]

SUPPLEMENTARY INFORMATION

belonging to the manuscript

Inter- and intracellular colonization of Arabidopsis roots by endophytic actinobacteria and the impact of plant hormones on their antimicrobial activity

Anne van der Meij[1, *], Joost Willemse[1,*], Martinus A. Schneijderberg[2], René Geurts[2], Jos M. Raaijmakers[3] and Gilles P. van Wezel[1, #]

[1] Molecular Biotechnology, Institute of Biology, Leiden University, Sylviusweg 72, 2333 BE, Leiden, The Netherlands.

[2] Department of Plant Sciences, Wageningen University, The Netherlands.

[3] Department of Microbial Ecology, Netherlands Institute of Ecology (NIOO-KNAW), Wageningen, The Netherlands.

Table S1. Number of replicates, sequence reads, total- and actinobacterial OTUs obtained with amplicon sequencing.

Number of:	soil	RS	EC	wood
replicates	8	8	8	8
av. sequence reads	282526	232525	31020	182009
av. OTUs	2664	2640	731	504
av. actinobacterial OTUs	82	81	51	33

RS: rhizosphere; EC: endophytic compartment.

Table S2. Antimicrobial activity of MOS isolates.

Activity against *E. coli*	MOS isolate #	Activity against *B. subtilis*	MOS isolate #
Strains with activity	16, 30, 35, 40	Strains with activity	23, 29, 30, 38, 40
increased activity SA	16	increased activity SA	22, 32, 38
increased activity JAS	none	increased activity JAS	8, 32, 38
increased activity IAA	16, 20, 29, 30, 37	increased activity IAA	16, 17, 30, 38

Red numbers represent strains that show no antibiotic activity under control conditions, but do when they are exposed to a phytohormone. Black numbers represent strains that show enhanced antimicrobial activity when grown in the presence of phytohormones. Green numbers are assigned to strains that were elicited by all phytohormones.

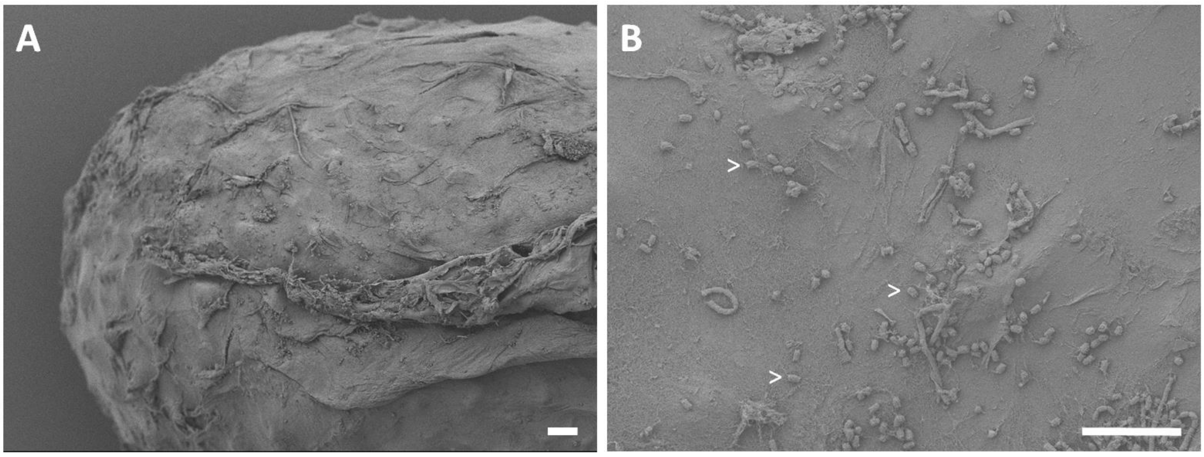

Figure S1. Attachment of spores to *Arabidopsis* seeds. A) Scanning electron micrograph of an *Arabidopsis* seed coat with spores. B) Zoom-in on seed coat. Individual spores are visible (arrowheads). Scale bars: 10 µM.

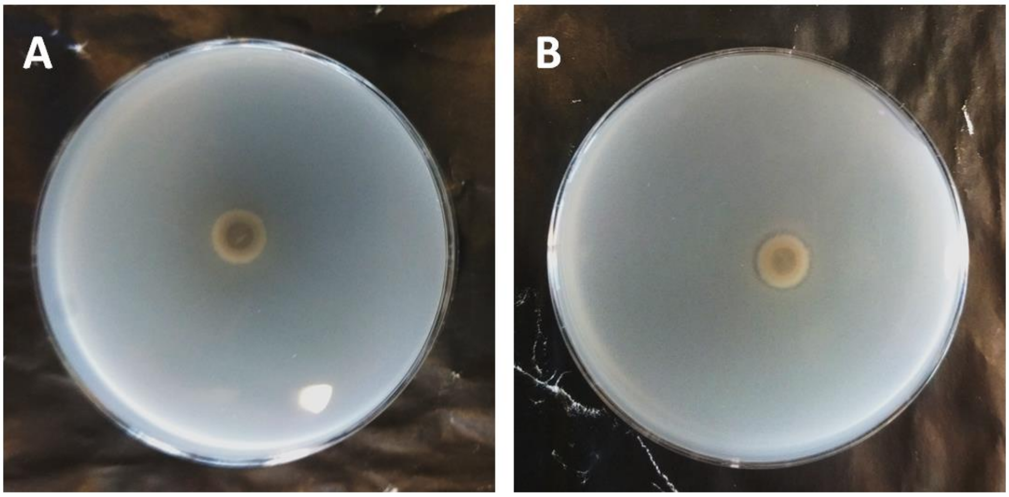

Figure S2. Antimicrobial activity of *Streptomyces* sp. MOS17 elicited by IAA. *Streptomyces* sp. MOS17 was brought on minimal medium without (A) or with (B) 0.001% IAA and grown for 5 days, followed by overlay with a lawn of *B. subtilis*. Note the small zone of growth inhibition just around the colony when IAA was added as elicitor.

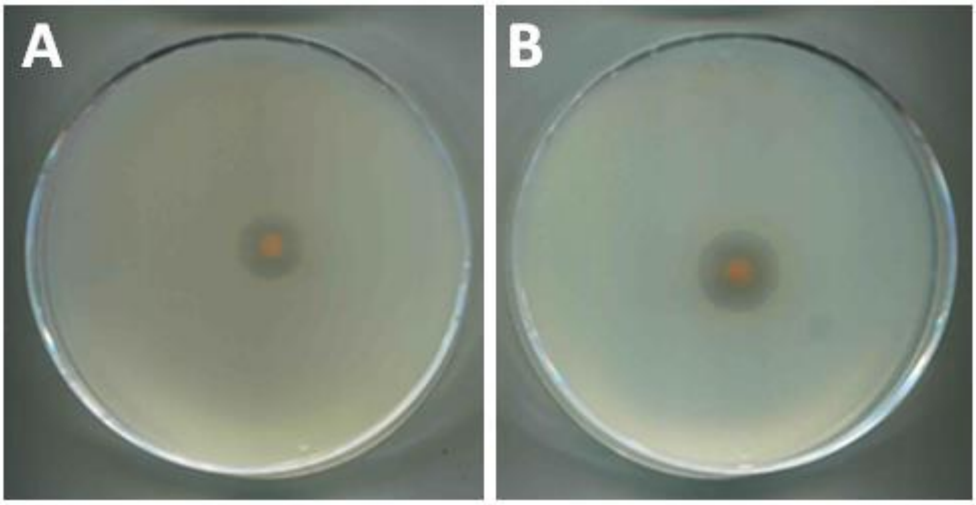

Figure S3. Elicitation of antimicrobial activity of ***Streptomyces*** **sp. MOS38 by IAA.** *Streptomyces* sp. MOS38 was brought on minimal medium without (A) or with (B) 0.01% IAA and grown for 5 days, followed by overlay with a lawn of *B. subtilis*. Note the increased zone of growth inhibition around the colony when IAA was added as elicitor.
